# Translating knowledge to practice: application of the public health apprenticeship

**DOI:** 10.3389/fpubh.2025.1632118

**Published:** 2025-06-23

**Authors:** Mary Elizabeth Pendergrass, Angela L. Carman

**Affiliations:** University of Kentucky College of Public Health, Lexington, KY, United States

**Keywords:** pedagogy, public health, apprenticeship, cognitive apprenticeship theory, public health practice

## Abstract

As public health challenges continue to grow and deepen in complexity, public health workforce roles are becoming increasingly difficult to fill. As a result, the public health educational system must adapt to address these dynamic workforce needs. Health-related professions commonly utilize experiential learning models such as post-graduate programs, practicums or applied practice experiences, apprenticeship, and mentorship. However, there is a gap in practice-based, post-master’s programs to develop these skills for Master of Public Health graduates. Accordingly, this paper proposes the public health apprenticeship, guided by the Cognitive Apprenticeship Theory, as a solution to fill this gap. A real-world application of the public health apprenticeship is described, and development of public health competencies are measured to evaluate the effectiveness of the public health apprenticeship model.

## 1 Introduction

Despite the ever-evolving health needs of the population, the public health workforce continues to dwindle (1). Leider et al. (1) review of the state of the public health workforce determined that the governmental public health workforce in the United States (U. S.) has lost 40,000 jobs between the 2009 recession and the COVID-19 pandemic. Further, public health roles are becoming increasingly difficult to fill, with a lack of availability of trained candidates applying for work in governmental public health agencies and an aging workforce (1, 2). As the public health workforce continues to change rapidly, the public health educational system must adapt to address this new landscape (3). The Association of Schools and Programs of Public Health’s (ASPPH) Framing the Future 2030 initiative calls on public health programs to utilize “transformative approaches to teaching and learning,” including experiential learning for hands-on skill development (3).

The Master of Public Health (MPH) is a practice-based degree wherein graduates are expected to translate classroom knowledge to real-world public health practice, ultimately filling a public health workforce role (4). MPH graduates are expected to be equipped with the knowledge and skills to perform essential public health functions, which requires exposure to real-world practice (4). Although MPH graduates are typically proficient in their classroom knowledge, prior research has established that MPH graduates often lack the skills necessary for an entry-level governmental public health position (5, 6). Further, the profession continues to evolve post-COVID, in regard to the scope, context, and landscape within which public health is practiced (2, 3, 7). Whereas experiential learning is a core component of public health curriculum, it is important to re-evaluate the MPH and its ability to prepare and equip proficient public health professionals to successfully enter the workforce (7, 8).

The Council on Education for Public Health is the public health accrediting body recognized by the United States Department of Education, currently accrediting 263 schools and programs, including a number of MPH programs (9). In an effort to incorporate knowledge translation and experiential learning into the MPH curriculum, all CEPH accredited programs require candidates to complete an applied practice experience, commonly known as a “practicum” (8). The intended purpose of a practicum is to build the capacity of MPH candidates by exposing them to the complex challenges of public health practice through real-world experience (10). However, the application of a practicum experience is not without its challenges, including great variation across non-accredited programs globally (10). Within CEPH-accredited programs, barriers to practicum placement persist (11). Although CEPH requires that the MPH practicum demonstrate attainment of 5 public health competencies, in practice many practicum experiences are not grounded in program concepts and may end up completely unrelated to the curriculum itself, leading to a lack of cohesion across learning experiences (8, 12).

One mechanism commonly utilized to incorporate experiential learning in health-related fields are post-graduate programs, commonly at the post-baccalaureate and post-doctoral levels (13). While post-baccalaureate programs are commonly focused on practice-based healthcare professions including nursing and medicine, post-doctoral programs traditionally focus on research (14, 15). The National Postdoctoral Association defines a postdoctoral scholar (postdoc) as, “an individual holding a doctoral degree who is engaged in a temporary period of mentored research and/or scholarly training for the purpose of acquiring the professional skills needed to pursue a career path of his or her choosing” (14). Although the length of post-graduate programs widely differs, previous research has established a positive relationship between the length of a fellowship and future scientific achievement, with each additional year of research training associated with a 21% increase in lifetime publications (16). Despite the many documented benefits of post-baccalaureate and post-doctoral programs, there is a gap in the literature regarding similar practice-based programs at the master’s level.

Another method commonly used to encourage skill development through experiential learning is mentorship (17). Documented benefits of mentorship are reciprocal and include individual growth, professional development, and psychosocial support (17). More broadly, mentorship is a type of relationship that enhances learning and skill-building, especially when coupled with apprenticeship learning (18). Apprenticeship is a form of learning wherein an apprentice observes an expert performing a task, practices under their supervision, and ultimately develops the skills to complete the task alone (19). This is one of the oldest forms of learning, predating formal education (19). Apprenticeship and mentorship are still commonly used across disciplines, together and separately, but still lack a pedagogical framework for development of necessary cognitive skills (18, 19).

While the goal of experiential learning methods such as public health practicum, post-graduate, and mentorship work remain the same: to equip graduates with the skills necessary to enhance and elevate their career, an adequate Master of Public Health learning experience is still lacking. This paper proposes the public health apprenticeship: a post-graduate, practice-based experience with a guided pedagogical framework as an innovative method through which to equip MPH graduates with the skills needed to impact public health.

## 2 Pedagogical framework and competencies

The Cognitive Apprenticeship Theory establishes a pedagogical framework through which apprenticeship bridges the many skill gaps present in formalized education, maximizing benefits from both traditional apprenticeship and mentorship (20). Specifically, the *cognitive* apprenticeship involves problem-solving utilizing not only factual knowledge, but also the context in which the problem exists, resulting in an emphasis on cognition rather than processes themselves (20). Cognitive Apprenticeship Theory combines teaching methods from the traditional and Cognitive Apprenticeship models seen in Table 1, as described by model authors Collins and Brown.

**Table 1:** Cognitive apprenticeship teaching methods.

Teaching method	Model derived from	Description
Modeling	Traditional apprenticeship model	“Involves an expert’s performing a task so that the students can observe and build a conceptual model of the processes required to accomplish it (20).”
Coaching	Traditional apprenticeship model	“Consists of observing students while they carry out a task and offering hints, scaffolding, feedback, modeling, reminders, and new tasks aimed at bringing their performance closer to expert performance (20).”
Scaffolding	Traditional apprenticeship model	“Refers to the supports the teacher provides to help the student carry out the task (20).”
Articulation	Cognitive apprenticeship model	“Involves any method of getting students to articulate their knowledge, reasoning, or problem-solving processes (20).”
Reflection	Cognitive apprenticeship model	“Involves enabling students to compare their own problem-solving processes with those of an expert, another student, and ultimately, an internal cognitive model of expertise (20).”
Exploration	Cognitive apprenticeship model	“Involves pushing students into a mode of problem solving on their own (20).”

Although prior literature does not describe application of Cognitive Apprenticeship in public health specifically, it has been successfully utilized with other allied health professions (21–24). Within health sciences education, cognitive apprenticeship has been applied in a variety of environments, from clinical settings to online coursework (21). One study measured the impact of Cognitive Apprenticeship Theory within pharmacological education, resulting in an increase in clinical reasoning scores and self-confidence (22). Further reviews focused on Cognitive Apprenticeship in clinical education found the model particularly effective for practice-based health professions, contributing to the development of both the student and faculty alike (23). Whereas the MPH is a practice-based health degree, it is logical that Cognitive Apprenticeship may be applied to public health similarly.

The public health apprenticeship utilizes the Cognitive Apprenticeship Model to develop and strengthen core public health competencies. Two key sets of public health competencies will be utilized to evaluate the learning impact of the public health apprenticeship. First, the Council on Linkages Between Academic and Public Health Practice (“Council on Linkages”) is an organization that sets forth and regularly revises competencies through which to assess the application of public health skills and concepts in real-world practice (25). In 2021, the Council on Linkages released their recently revised set of competencies, including 8 domains, which will later be used to measure competency development of the public health apprenticeship (25):

1. Data Analytics and Assessment Skills
2. Policy Development and Program Planning Skills
3. Communication Skills
4. Health Equity Skills
5. Community Partnership Skills
6. Public Health Sciences Skills
7. Management and Finance Skills
8. Leadership and Systems Thinking Skills

Similarly, CEPH sets forth foundational competencies based on required curricula to be demonstrated by MPH graduates, which will be utilized to evaluate the impact of the public health apprenticeship (8). CEPH’s foundational MPH competencies are organized into the following categories:

1. Evidence-based Approaches to Public Health
2. Public Health & Health Care Systems
3. Planning & Management to Promote Health
4. Policy in Public Health
5. Leadership
6. Communication
7. Interprofessional and/or Intersectoral Practice
8. Systems Thinking

## 3 Learning environment

Team Up: a Public Health Academic Practice Collaborative, supported by the Foundation for a Healthy Kentucky, hosted the public health apprentice. Team Up is an initiative developed in 2023 and led by community-facing faculty at the University of Kentucky College of Public Health that aims to bridge the gap between academia and practice in public health through collaborative community organizing, policy and planning decision support, and applied scholarship. During its formation, Team Up identified the need for increased experiential education for MPH students, which paired perfectly with the support service delivery projects faculty were already working on. As a result of relationships established by community-facing faculty, Team Up students were able to complete a variety of projects such as strategic planning, secondary data collection and presentation, and community health assessment and improvement planning. As Team Up expanded, its partnership network also grew extensively to include several local health departments, healthcare organizations, hospital systems, public health leadership associations, and even a formalized academic health department. This growth, coupled with the lessons learned from hosting 4 MPH practicum students, led faculty to consider the potential impact of employing one recent graduate to complete this work full-time for 1 year, similar to the aforementioned post-graduate learning strategies. As a result, faculty selected a recent MPH graduate to employ full-time (40 hours per week) for one year as the public health apprentice.

Due to the diversity of partnerships and projects, the physical learning environment of the apprentice was varied. The apprentice travelled to meet and work with community partners frequently, oftentimes meeting at local health departments, hospital facilities, and event venues. Collaboration with multiple community partners on multiple projects contributed to an ideal learning environment for the apprentice, allowing for a wide variety of experiences and opportunities for skill development. While not travelling, the apprentice work was completed in a hybrid format, working remotely about 60% of the time, and on campus at the University of Kentucky the remaining 40%. Remote work tasks involved a variety of computer-based work, including writing, virtual meetings, training, and material preparation. Additionally, the apprentice met weekly with the preceptor and Team Up students to share updates and collaborate on projects, meeting a total of 39 times during the apprenticeship year (Spring 2024-Spring 2025).

The Team Up lead and community-facing faculty member served as the preceptor for the present apprenticeship. With almost two decades of public health practice experience, the preceptor’s service to the field is conducted through support service delivery, workforce development for practicing public health staff, and accreditation readiness for governmental health departments seeking national public health accreditation. Their community-facing work, extensive public health education, contributions to the field, and mentorship capabilities made them an ideal candidate to be the apprenticeship preceptor.

Throughout the variety of projects the apprentice worked on throughout the year, the Cognitive Apprenticeship Teaching Methods were applied to expedite and enhance learning. One such project was a contracted Community Health Needs Assessment (CHNA) with a hospital group operating 14 facilities in Eastern Kentucky and West Virginia. The CHNA project involved 60 + community forums, 4,000 + surveys, and compilation and analysis of qualitative, quantitative, primary, and secondary data for each of the 14 hospital facilities. The preceptor applied teaching methods from the Cognitive Apprenticeship Theory to enhance learning through the CHNA project. See Table 2 for an example of how each teaching method was implemented.

**Table 2:** Cognitive apprenticeship theory application in community health needs assessment project.

Teaching method (20)	Application
Modeling	Preceptor prepared the materials for and led the first community health forum, apprentice observed.
Coaching	Apprentice prepared materials for second community health forum, preceptor critiqued.
Scaffolding	Preceptor sent apprentice to a facilitation training to equip them with the skills needed to conduct the community health forums.
Articulation	Apprentice led the remainder of the 60 + community health forums alone.
Reflection	Toward the end of the forums, apprentice and preceptor discussed what they would do differently next time after seeing the process through.
Exploration	Preceptor allowed apprentice to become main point of contact for the project, apprentice utilized data analysis and problem-solving skills to analyze qualitative and quantitative data and prepare final reports.

## 4 Results to date

**Figure 1:**
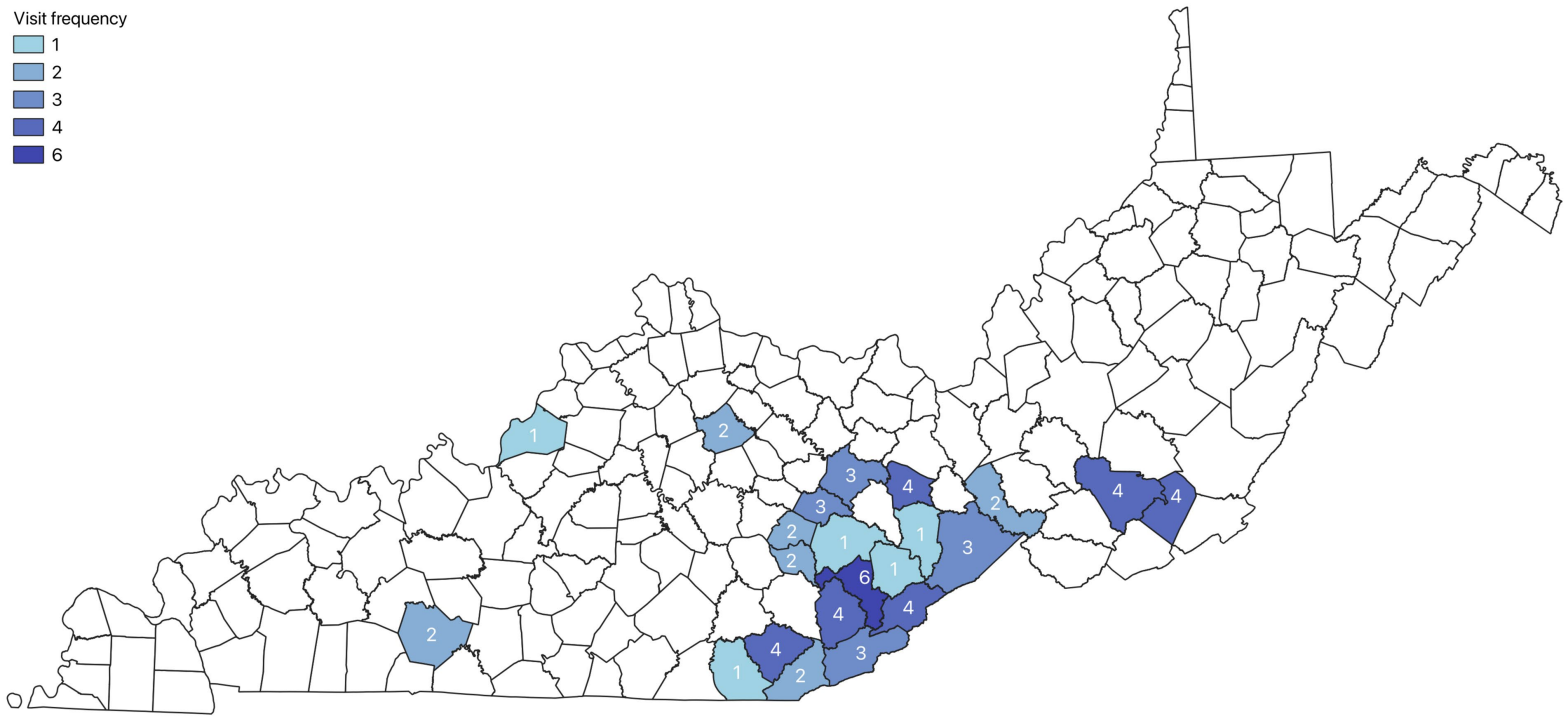
Apprentice travel frequency map.

To demonstrate the extent of work done with public health practice partners external to the University, miles travelled during the apprenticeship year were documented. From May 5, 2024, to May 5, 2025, the apprentice travelled over 14,000 miles completing trainings, advocacy, and facilitating community partner meetings. See Figure 1 for a map of apprenticeship travel within Kentucky and West Virginia.

To evaluate the learning impact of the apprenticeship, activities and projects were mapped to competencies from CEPH and the Council on Linkages Between Academia and Public Health Practice. The Council on Linkages Between Academia and Public Health Practice (Council on Linkages) sets forth competencies representing the foundational skills needed to enter the public health workforce (25). Similarly, CEPH sets forth foundational competencies that MPH graduates must demonstrate. Projects completed during the apprenticeship year (May 2024–May 2025) were listed under each domain and sub-domain to demonstrate fulfillment of each competency. Table 3 includes a sample of competency-fulfilling projects, see supplementary material for the full analysis tables.

**Table 3:** Sample of apprenticeship projects satisfying public health competencies.

Organization	Domain	Competency	Project fulfillment
Council on linkages	1: Data analytics and assessment skills	*1.8 Assesses community health status*	CHNA for a 14-facility rural healthcare system
Council on linkages	2: Policy development and program planning skills	*2.3 Evaluates policies, programs, services, and organizational performance*	School-based smoke free signage evaluation
Council on linkages	3: Communication skills	*3.4 Facilitates communication among individuals, groups, and organizations*	Facilitation of an 8-county, regional health consortium
Council on linkages	4: Health equity skills	*4.6 Contributes to achieving and sustaining a diverse, inclusive, and competent public health workforce*	Participated in training for MAPP 2.0 to develop health equity skills for incorporation in community health improvement projects.
Council on linkages	5: Community partnership skills	*5.2 Establishes relationships to improve community health and resilience*	Regular meetings with 7-county local health department district designated as an academic health department
Council on linkages	6: Public health sciences skills	*6.4 Contributes to the evidence base for improving health*	Poster presentation: regional professional group meeting
Council on linkages	7: Management and finance skills	*7.10 Applies critical thinking in decision-making*	Amending the schedule for projects involving counties who experienced natural disasters and/or traumatic events
Council on linkages	8: Leadership and systems thinking skills	*8.1 Creates opportunities to achieve cross-sector alignment*	Development of Local Public Health Schematics utilizing county-specific data to highlight potential consortium members and partnerships
CEPH	9: Evidence-based approaches to public health	*4: Interpret results of data analysis for public health research, policy, or practice*	Interpretation of quantitative survey data and qualitative focus group data and presentation to steering committee groups
CEPH	Planning and management to promote health	*7: Assess population needs, assets, and capacities that affect communities’ health*	Facilitation and documentation of local health department strategic plan
CEPH	Communication	*18: Select communication strategies for different audiences and sectors*	Selecting quantity of secondary data to present to consortium focus groups

## 5 Discussion and implications

This example of the public health apprenticeship suggests that the Cognitive Apprenticeship Theory is a successful pedagogical model for development of core public health competencies. For the apprentice, the opportunity to learn by observing the preceptor complete practice-based tasks and subsequently performing those tasks themself builds confidence in practice-based skills. This method also allows the apprentice to see public health in ways that are very difficult to replicate in the classroom. For example, the travel involved in the CHNA project made the distance between health facilities, full-service grocery stores, impact of community water problems, and challenges with housing and transportation real and left the apprentice with the ability to better understand situations community members encounter on a daily basis. Moreover, interacting with practitioners and those personally impacted by the daily struggle to obtain basic needs adds a layer of meaning and understanding of the impact and importance of public health work, contributing to personal and professional growth. Having completed many tasks during the apprenticeship, it is the authors belief that the apprentice is better prepared to be an integral part of any public health organization. In addition, the practice-based activities completed by both the apprentice and the preceptor further expands the reach of academia as a true partner to those community members working to improve health every day.

For academic organizations interested in replicating the public health apprenticeship, the academic hosting institution must have knowledge of and established relationships with public health practitioners. These relationships can be developed both through research projects and separately through support service delivery, professional organization attendance, among other means. Similarly, the institution must have community-facing faculty willing to serve as the preceptor who is able to conduct apprentice teaching through the methods set forth in the Cognitive Apprenticeship Theory. This requires a significant time investment and is increasingly feasible if the preceptor is already working on public health practice projects that could include the apprentice. Importantly, the public health apprenticeship represents a culmination of the benefits of the learning methods traditionally utilized in health profession education, including practicum, post-graduate programs, mentorship, and apprentice learning. Additionally, it is important to secure a funding source to support the practice-based apprentice work. The present apprentice received a salary determined by industry standard for recent MPH graduates, funded by the preceptor’s endowment and support service delivery funding streams. Similarly, the preceptor funding streams compensated the apprentice for miles travelled to complete partner work. The projects and travel completed by the apprentice recouped large amounts of time for the preceptor to further their work on other fronts, increasing preceptor capacity to publish and present completed work, provide mentorship to additional students, and strengthen and sustain relationships with community partners.

Due to the demonstrated lack of practice-based, post-master’s programs, the need for such type of education is increasing. Although experiential learning initiatives such as practicum have been integrated into Master of Public Health programs, utilizing the public health apprenticeship provides additional benefits to experiential learning and professional development (26). Further, post-graduate training programs at non-academic institutions such as the Centers for Disease Control and Prevention (CDC) and the Council of State and Territorial Epidemiologists lack the pedagogical framework necessary for the transition from academia to practice, resulting in a similar workforce training gap. During a time when the public health workforce is dwindling and undertrained, it is imperative for public health academia to develop and train a diverse and skilled workforce (1, 3). Utilizing the public health apprentice may be a solution.

## 6 Limitations

It is important to acknowledge that the present apprenticeship was conducted as a pilot initiative through an established program of collaboration with academia and practice, Team Up: A Public Health Academic Practice Collaborative. Future replication of the public health apprenticeship may establish additional benefits, lessons, and implications. Finally, many of the skills gained through the apprenticeship are difficult to capture or quantify, but are invaluable to public health practice (e.g., resilience).

## 7 Recommendations

Given the need for innovative public health workforce development strategies, authors recommend further application of the public health apprenticeship through academic institutions. Importantly, academic public health institutions should prioritize the presence of community-facing faculty in order to cultivate a similar learning environment to the present apprenticeship. Ensuring faculty are engaged with both the community and practice partners is an essential component of fostering experiential learning (27). Additionally, authors recommend academic integration of the apprenticeship in a similar manner to that of the post-doctoral fellow. In this manner, the apprentice may apply knowledge from their completion of an MPH program in real-world practice. In consideration of funding, authors recommend reviewing funding streams that may already be utilized to complete faculty research and service work. As the apprentice is employed post-graduation, in a similar manner to a post-doctoral student, institutions replicating the public health apprenticeship may also consider similar funding opportunities to post-doctoral work, including but not limited to grant funding, fellowships, and preceptor-funded work.

As the present assessment demonstrates, application of the Cognitive Apprenticeship Theory is an effective method through which MPH graduates may develop the cognitive skills necessary to enter the public health workforce. Through repeated exposure to real-world public health practice, within the safety of the academic preceptor, graduates who complete the apprenticeship are equipped with the confidence and comfortability necessary to confidently enter the governmental public health workforce. In order to impact the public health workforce on a larger scale, authors recommend that additional academic institutions adopt the public health apprenticeship. This increased number of fully equipped MPH apprenticeship graduates will greatly impact the public health workforce gap.

## Data Availability

The original contributions presented in the study are included in the article/Supplementary material, further inquiries can be directed to the corresponding author.
