# Supplementary material for: Translating knowledge to practice: application of the public health apprenticeship
Source: Front Public Health. 2025 Jun 23;13:1632118. doi: 10.3389/fpubh.2025.1632118 (PMC12230075; doi:10.3389/fpubh.2025.1632118)
Supplement: Supplementary file 1 [file Table_1.docx]

Supplementary Material

# Supplementary Table 1

Evaluation of Apprenticeship through Council on Linkages Public Health Competencies(16)

| **Domain 1: Data Analytics and Assessment Skills** | | | | | |
| --- | --- | --- | --- | --- | --- |
| 1.1 Describes factors that affect the health of a community | SDOH Infographics | CHA/CHIP poster |  |  |  |
| 1.2 Access existing quantitative and qualitative data | SDOH Infographics | Secondary data collection |  |  |  |
| 1.3 Collects quantitative and qualitative data | CHNA for a 14-facility rural healthcare system | Single County LHD CHA/CHIP | Single County LHD Strategic Plan | Single County LHD CHA/CHIP #2 |  |
| 1.4 Analyzes quantitative and qualitative data | CHNA for a 14-facility rural healthcare system | Single County LHD CHA/CHIP | Single County LHD CHA/CHIP |  |  |
| 1.5 Manages quantitative and qualitative data | CHNA for a 14-facility rural healthcare system | Single County LHD CHA/CHIP | Single County LHD CHA/CHIP #2 | Single County LHD Strategic Plan |  |
| 1.6 Uses quantitative and qualitative data | CHNA for a 14-facility rural healthcare system | Single County LHD CHA/CHIP | Single County LHD CHA/CHIP | Single County LHD Strategic Plan |  |
| 1.7 Applies public health informatics in using data, information, and knowledge | AI random selection-smoke free evaluation | Single County LHD CHA/CHIP dashboard |  |  |  |
| 1.8 Assesses community health status | CHNA for a 14-facility rural healthcare system | Single County LHD CHA/CHIP | Single County LHD CHA/CHIP #2 |  |  |
| **Domain 2: Policy Development and Program Planning Skills** | | | | | |
| 2.1 Develops policies, programs, and services | Single County LHD QI manual | Weekly Team Updates | Academic Health Department |  |  |
| 2.2 Implements policies, programs, and services | Pulling/ disseminating Evidence based practice | Academic Health Department- regular meeting |  |  |  |
| 2.3 Evaluates policies, programs, services, and organizational performance | Team Up strategic plan | Single county LHD strategic plan | Smoke free school evaluation |  |  |
| 2.4 Improves policies, programs, services, and organizational performance | Revised 3 core areas: Team Up | Single County LHD Quality Improvement training |  |  |  |
| 2.5 Influences policies, programs, and services external to the organization | National conference policy institute- advocacy |  |  |  |  |
| 2.6 Engages in organizational strategic planning | Team Up strategic plan | Single County LHD strategic plan |  |  |  |
| 2.7 Engages in community health improvement planning | CHNA for a 14-facility rural healthcare system | Single County LHD CHA/CHIP | Single County LHD CHA/CHIP #2 |  |  |
| **Domain 3: Communication Skills** | | | | | |
| 3.1 Determines communication strategies | Team Updates | Academic Health Department- Regular Meeting |  |  |  |
| 3.2 Communicates with internal and external audiences | Facilitation- CHNA for a 14-facility rural healthcare system | Facilitation with an 8-county, regional health consortium | Academic Health Department- research project coordination | Poster presentation at academic conference |  |
| 3.3 Responds to information, misinformation, and disinformation | Literature reviews | Sensemaking- students | Sensemaking- students | Immunization conference |  |
| 3.4 Facilitates communication among individuals, groups, and organizations | Facilitation- CHNA for a 14-facility rural healthcare system | Facilitation with an 8-county, regional health consortium | Academic Health Department- coordination of service project |  |  |
| **Domain 4: Health Equity Skills** | | | | | |
| 4.1 Applies principles of ethics, diversity, equity, inclusion, and justice | SDOH Infographics | Equity in facilitation |  |  |  |
| 4.2 Engages in continuous self-reflection about one's biases | Write-ups after trips |  |  |  |  |
| 4.3 Recognizes the diversity of individuals and populations | Change style indicator |  |  |  |  |
| 4.4 Reduces systemic and structural barriers that perpetuate health inequities | SDOH Infographics | Custom Local Public Health schematics | Meeting partners where they are |  |  |
| 4.5 Implements organizational policies, programs, and services to achieve health equity and social and environmental justice | Team Updates design and implementation | Facilitation training- applying health equity concepts in CHNA focus groups |  |  |  |
| 4.6 Contributes to achieving and sustaining a diverse, inclusive, and competent public health workforce | Single County LHD Quality Improvement training | MAPP 2.0 training | Facilitation training | Photovoice training |  |
| 4.7 Advocates for health equity and social and environmental justice | National conference policy institute- advocacy |  |  |  |  |
| **Domain 5: Community Partnership Skills** | | | | | |
| 5.1 Describes conditions, systems, and policies affecting community health and resilience | CHNA for a 14-facility rural healthcare system | SDOH infographics | 8-County Health Consortium CHA/CHIP |  |  |
| 5.2 Establishes relationships to improve community health and resilience | National conference policy institute : Kentucky contingent | Academic Health Department | CHNA steering committees |  |  |
| 5.3 Maintains relationships that improve community health and resilience | Academic Health Department Christmas party | Academic Health Department summer celebration/ activity | Coordination of Academic Health Department service project |  |  |
| 5.4 Collaborates with community members and organizations | County health coalition | County health coalition #2 | 8-county regional health consortium | CHNA steering committees |  |
| 5.5 Shares power and ownership with community members and others | Facilitation- CHNA for a 14-facility rural healthcare system | County health coalition | 8-county regional health consortium |  |  |
| **Domain 6: Public Health Sciences Skills** | | | | | |
| 6.1 Describes systems, policies, and events impacting public health | Sharing public health in news articles | Discussing policy changes with healthcare partners | Discussing policy changes with academic health department | Economic aspect of Medicaid expansion |  |
| 6.2 Applies public health sciences in delivering the 10 Essential Public Health Services | Using evidence-based models (MAPP) | Health Behavior theory application | Dissemination of Innovations- smoke free schools evaluation |  |  |
| 6.3 Uses evidence in developing, implementing, evaluating, and improving policies, programs, and services | MAPP model in CHA/CHIP | Pulling evidence-based practice for healthcare partners | Pulling evidence-based practice for academic health department |  |  |
| 6.4 Contributes to the evidence base for improving health | MAPP 2.0 paper | Building trust through service delivery | Professional regional conference- poster presentation |  |  |
| **Domain 7: Management and Finance Skills** | | | | | |
| 7.1 Describes factors that affect the health of an organization | Team Up project collaboration | Change style indicator |  |  |  |
| 7.2 Secures human resources | Student searching | Internship qualifications sheets |  |  |  |
| 7.3 Manages human resources | Team Updates | Assigning lead role to projects |  |  |  |
| 7.4 Engages in professional development | Facilitation training | MAPP 2.0 training | Photovoice training | Online QI training | Immunization conference |
| 7.5 Secures financial resources | Completion of contracted services |  |  |  |  |
| 7.6 Manages financial resources | Travel arrangements- DC | Travel arrangements- Dallas | Travel arrangements- CHNA |  |  |
| 7.7 Implements organizational policies, programs, and services to achieve diversity, equity, inclusion, and justice | Book club |  |  |  |  |
| 7.8 Manages programs and services | Team Updates | Assigning lead role to projects |  |  |  |
| 7.9 Engages in contingency planning | Team Updates | Book club |  |  |  |
| 7.10 Applies critical thinking in decision making | Travelling during bad weather | Preparing materials for KRHC- cultural competence | Cancelling meetings after community experienced trauma |  |  |
| 7.11 Engages individuals and teams to achieve program and organizational goals | Team Up strategic plan |  |  |  |  |
| 7.12 Facilitates collaboration among individuals, groups, and organizations | Academic showcase poster preparation- peer reviews |  |  |  |  |
| 7.13 Engages in performance management | Matching activities to competencies |  |  |  |  |
| **Domain 8: Leadership and Systems Thinking Skills** | | | | | |
| 8.1 Creates opportunities to achieve cross-sector alignment | Local Public Health Schematics |  |  |  |  |
| 8.2 Implements a vision for a healthy community | Team Up vision statement | CHNA for a 14-facility rural healthcare system |  |  |  |
| 8.3 Addresses facilitators and barriers impacting delivery of the 10 Essential Public Health Services | Professional regional conference- poster presentation | Academic Health Dept |  |  |  |
| 8.4 Creates opportunities for creativity and innovation | Office bulletin board | Academic health department- service project | Public Health Halloween |  |  |
| 8.5 Responds to emerging needs | Quick turnaround on emergent projects | Flood response- infographic sharing |  |  |  |
| 8.6 Manages organizational change | Team Up organizational change- re-assigned roles and responsibilities | Change style indicator | County LHD QI training |  |  |
| 8.7 Engages politicians, policymakers, and the public to support public health infrastructure | National conference policy institute- advocacy | Sharing support to focus groups | Educating non-PH focus groups on PH |  |  |
| 8.8 Advocates for public health | National conference policy institute- advocacy |  |  |  |  |

# Supplementary Table 2

Evaluation of Apprenticeship through CEPH MPH Foundational Competencies

| **Evidence-based Approaches to Public Health** | | | | |
| --- | --- | --- | --- | --- |
| Apply epidemiological methods to settings and situations in public health practice | Secondary data collection | SDOH infographics | CHA/CHNA data analysis |  |
| Select quantitative and qualitative data collection methods appropriate for a given public health context | Smoke-free school evaluation | Quantitative. &qualitative data collection- CHNA for a 14-facility rural healthcare system | 8-County Health Consortium CHA/CHIP |  |
| Analyze quantitative and qualitative data using biostatistics, informatics, computer-based programming and software, as appropriate | Focus group thematic analysis | Survey software data analysis |  |  |
| Interpret results of data analysis for public health research policy or practice | CHNA steering committee meetings- interpret and present findings | Research poster presentation at academic conference |  |  |
| **Public Health & Health Care Systems** | | | | |
| Compare the organization, structure, and function of health care, public health, and regulatory systems across national and international settings | Research poster presentation at academic conference |  |  |  |
| Discuss the means by which structural bias, social inequities and racism undermine health and create challenges to achieving health equity at the organizational, community, and systemic levels | SDOH infographics | CHNA steering committee meetings |  |  |
| **Planning & Management to Promote Health** | | | | |
| Assess population needs, assets, and capacities that affect communities’ health | CHNA focus groups | Local health department strategic plan | Local health department CHA/CHIP |  |
| Apply awareness of cultural values and practices to the design, implementation, or critique of public health policies or programs | Trauma-informed facilitation techniques | Natural disaster relief cards | Meeting partners where they are |  |
| Design a population-based policy, program, project, or intervention | Natural disaster relief cards |  |  |  |
| Explain basic principles and tools of budget and resource management | Travel arrangements- CHNA | Travel arrangements- trainings |  |  |
| Select methods to evaluate public health programs | Smoke-free evaluation | Development of prospective evaluation plan and documentation for academic health department |  |  |
| **Policy in Public Health** | | | | |
| Discuss the policy-making process, including the roles of ethics and evidence | National conference policy institute- advocacy | Policy book club |  |  |
| Propose strategies to identify relevant communities and individuals to build coalitions and partnerships for influencing public health outcomes | 8-county regional health consortium | Academic health department |  |  |
| Advocate for political, social, or economic policies and programs that will improve health in diverse populations | Advocacy- national rural policy institute | Basic needs identification- CHNA |  |  |
| Evaluate policies for their impact on public health and health equity | Smoke-free school evaluation |  |  |  |
| **Leadership** | | | | |
| Apply leadership and/or management principles to address a relevant issue | Application of management models in Team Up strategic planning and core development |  |  |  |
| Apply negotiation and mediation skills to address organizational or community challenges | Facilitation training |  |  |  |
| **Communication** | | | | |
| Select communication strategies for different audiences and sectors | Data selection and presentation for focus groups versus steering committees | Development of quality improvement manual |  |  |
| Communicate audience-appropriate public health content, both in writing and through oral presentation to non-academic, non-peer audience with attention to factors such as literacy & health literacy | Focus group material development and presentation | Regional health conference poster presentation |  |  |
| Describe the importance of cultural humility in communicating public health content | Student sensemaking | Data selection and presentation |  |  |
| **Interprofessional and/or Intersectoral Practice** | | | | |
| Integrate perspectives from other sectors and/or professionals to promote and advance population health | Custom local public health schematic development |  |  |  |
| **Systems Thinking** | | | | |
| Apply a systems thinking tool to visually represent a public health issue in a format other than a standard narrative | Health behavior theory and model application | Systems thinking model application- asset mapping |  |  |

**
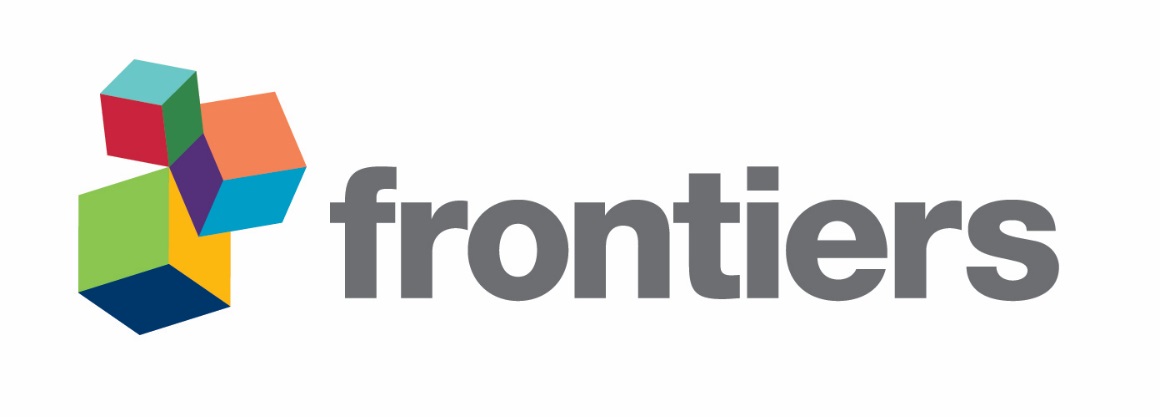
**
